# In Memoriam – Honouring Dr. Don Wigle

**DOI:** 10.24095/hpcdp.46.6.04

**Published:** 2026-06

We would like to acknowledge the passing of Don Wigle, MD, PhD, MPH, who died on February 19, 2026, in Ottawa. In 1980, Don was the impetus for the creation of a new journal, *Chronic Diseases in Canada*. The journal was renamed *Chronic Diseases and Injuries in Canada* in 2011 and *Health Promotion and Chronic Disease Prevention in Canada* in 2015. Don saw the establishment of the journal as a key mechanism to highlight the research and surveillance activities of the Laboratory Centre for Disease Control (a precursor of the Public Health Agency of Canada) and of other Canadian health researchers. Don was the principal investigator of many epidemiological studies of environmental hazards, including studies of radon and pesticides, and was a fearless champion for government action against cigarette smoking.

